# Neuroanatomical correlates of peripersonal space: bridging the gap between perception, action, emotion and social cognition

**DOI:** 10.1007/s00429-024-02781-9

**Published:** 2024-04-29

**Authors:** Gianpaolo Antonio Basile, Elisa Tatti, Salvatore Bertino, Demetrio Milardi, Giovanni Genovese, Antonio Bruno, Maria Rosaria Anna Muscatello, Rosella Ciurleo, Antonio Cerasa, Angelo Quartarone, Alberto Cacciola

**Affiliations:** 1 Brain Mapping Lab, Department of Biomedical, Dental Sciences and Morphological and Functional Imaging, University of Messina, Messina, Italy; 2 Department of Molecular, Cellular & Biomedical Sciences, CUNY, School of Medicine, New York, NY 10031 USA; 3 Department of Clinical and Experimental Medicine, University of Messina, Messina, Italy; 4 Psychiatry Unit, University Hospital “G. Martino”, Messina, Italy; 5 Department of Biomedical, Dental Sciences and Morphological and Functional Imaging, University of Messina, Messina, Italy; 6 IRCCS Centro Neurolesi “Bonino Pulejo”, Messina, Italy; 7 S. Anna Institute, Crotone, Italy; 8 Institute for Biomedical Research and Innovation (IRIB), National Research Council of Italy, Messina, Italy; 9 Pharmacotechnology Documentation and Transfer Unit, Preclinical and Translational Pharmacology, Department of Pharmacy, Health Science and Nutrition, University of Calabria, Rende, Italy

**Keywords:** Anatomy, Attention, Function, Networks, Neuroimaging, Neuropsychiatry

## Abstract

Peripersonal space (PPS) is a construct referring to the portion of space immediately surrounding our bodies, where most of the interactions between the subject and the environment, including other individuals, take place. Decades of animal and human neuroscience research have revealed that the brain holds a separate representation of this region of space: this distinct spatial representation has evolved to ensure proper relevance to stimuli that are close to the body and prompt an appropriate behavioral response. The neural underpinnings of such construct have been thoroughly investigated by different generations of studies involving anatomical and electrophysiological investigations in animal models, and, recently, neuroimaging experiments in human subjects. Here, we provide a comprehensive anatomical overview of the anatomical circuitry underlying PPS representation in the human brain. Gathering evidence from multiple areas of research, we identified cortical and subcortical regions that are involved in specific aspects of PPS encoding.

We show how these regions are part of segregated, yet integrated functional networks within the brain, which are in turn involved in higher-order integration of information. This wide-scale circuitry accounts for the relevance of PPS encoding in multiple brain functions, including not only motor planning and visuospatial attention but also emotional and social cognitive aspects. A complete characterization of these circuits may clarify the derangements of PPS representation observed in different neurological and neuropsychiatric diseases.

## Introduction

Over time, the human brain has developed the ability to categorize perceptual inputs into a unified, self-centered frame of reference for organizing spatial information. Extensive clinical and experimental research has shown that the brain encodes spatial information in three distinct manners: personal space, which corresponds to the surfaces within and outside the body; extrapersonal space, referring to the area farther away from the body; and peripersonal space (PPS), which encompasses the region where all physical interactions between the individual and the surroundings occur (Serino 2019). These three distinct definitions of space are represented separately in the primate brain (Rizzolatti et al. 1983; Cléry et al. 2018), and patients with focal brain lesions who experience spatial neglect exhibit clinically distinguishable impairments in each of these spatial domains (Beschin and Robertson 1997).

Cognitive neurosciences have shown great interest in investigating the neural mechanisms involved in encoding the PPS in the human brain. This interest stems from the fact that PPS encoding relies heavily on integrating multisensory information sources (Macaluso and Maravita 2010) and is essential for multiple higher-order cognitive functions (Graziano and Cooke 2006; Pellencin et al. 2018a; Serino 2019).

PPS is defined as the space where the human body interacts with the surrounding environment, making bodily representation a crucial component. Consequently, one of the core neural mechanisms underlying PPS representation is the remapping of visual information into body-centered coordinates such as head-centered, limb-centered, or trunk-centered coordinates(Colby 1998; Pouget et al. 2002; Avillac et al. 2005). The neural representation of the body and its immediate surroundings is so deeply interconnected that the construct of PPS bears substantial similarities and overlaps with the concept of body schema in psychophysiological terms (Cardinali et al. 2009). Moreover, recent studies suggest that the conscious experience of having a body, known as body ownership (Crucianelli et al. 2023), may depend on multisensory integration processes involved in PPS representation (Botvinick and Cohen 1998; Blanke and Metzinger 2009; Blanke 2012; Grivaz et al. 2017).

The encoding of objects in the PPS is key for complex movement planning, including reaching, grasping, and manipulation, which requires a strict interaction between the neural systems involved in PPS representation and the premotor system (Jeannerod et al. 1995; Rizzolatti et al. 1997; Gardner et al. 2007). This interaction is dynamic and bidirectional, as evidenced by the fact that movements like grasping or walking can induce adaptations in PPS extension around the body parts involved in those movements (Brozzoli et al. 2009, 2010; Noel et al. 2015). Additionally, the use of tools has been shown to dynamically expand PPS representation boundaries. This adaptive response is use-dependent and transient: the spatial representation has been shown to expand only if the tool is actively used (rather than passively held), and “shrinks back” to the original size after the end of tool use. Put differently, during their use, tools are incorporated as a part of the body schema, and the space surrounding the tools is processed as surrounding the subject(lriki et al. 1996; Farnè and Làdavas 2000; Farnè et al. 2005).

Behavioral studies have provided evidence that visual attention is enhanced for stimuli located close to the hands, suggesting that encoding of objects in the PPS may facilitate grasping and drive visuospatial attention towards objects that are within immediate reach (Reed et al. 2006; Dufour and Touzalin 2008; Brockmole et al. 2013). This idea has been extended to propose that PPS representation has evolved to enhance the perceptual salience of objects within proximity to the body, which can then be identified as immediately relevant for interaction, suggesting a central role for PPS representation in visuospatial attention (Thomas and Sunny 2017).

In addition to its role in grasping and motor planning, the representation of stimuli in the PPS also requires a heightened attentional response to prepare for and coordinate defensive responses against unwanted intruders. When objects rapidly enter the PPS, such as looming objects, they trigger an immediate attentional response due to their potential as a threat to the subject, immediately activating adaptive mechanisms required for avoidance and defensive behavior (Graziano and Cooke 2006). This concept of PPS as a “safety margin” around the body highlights the interaction between sensorimotor integration and the neural systems involved in emotional and behavioral responses to stressors and threats (Sambo et al. 2012; Hunley and Lourenco 2018). As such, it is not surprising that emotional states, such as anxiety, or trait psychological factors, such as personality traits, can influence the boundaries of PPS representation (Sambo and Iannetti 2013a; Fossataro et al. 2016; Hunley et al. 2017; Spaccasassi and Maravita 2020; Ellena et al. 2022). The close relationship between PPS representation and emotional processing explains why PPS is also relevant for social interactions. Recently, there has been a growing interest in studying the social implications of PPS representation (Heed et al. 2010; Bogdanova et al. 2021). Indeed, studies have shown that the presence of other individuals can modify the extension of PPS, and their perceived moral character, hostility, or cooperativeness can influence the size of PPS (Teneggi et al. 2013; Ruggiero et al. 2017; Cartaud et al. 2018; Pellencin et al. 2018b). These findings suggest a strong influence of the “social brain” (Adolphs 2009) on the multisensory integration underlying PPS representation.

**Fig. 1:**
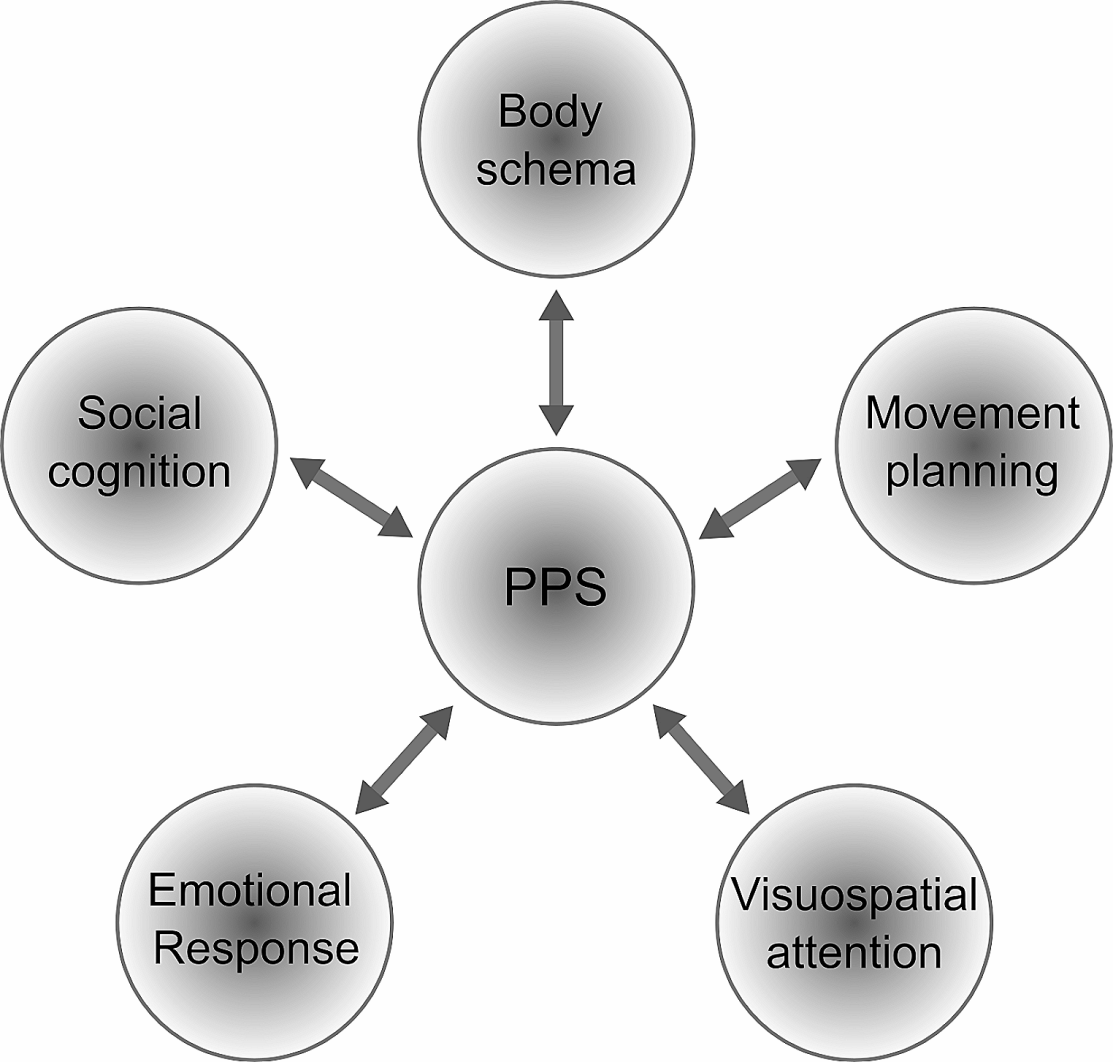
PPS representation as a bridge between psychobiological domains. The diagram emphasizes the relevance of PPS representation as a link between different brain functions

To summarize, the concept of PPS serves as a crucial bridge linking various domains of brain function, including spatial perception, self-perception, body schema, motor planning, visuospatial attention, emotional and social cognition (Fig. 1). Furthermore, alterations in PPS representations have been observed in numerous clinical conditions, from brain lesion-related neuropsychological syndromes(Ten Brink et al. 2019; Bassolino et al. 2022) to psychiatric disorders like anxiety, schizophrenia, and autism (Taffou and Viaud-Delmon 2014; Noel et al. 2017; Di Cosmo et al. 2018; Lee et al. 2021a). Therefore, a comprehensive understanding of the functional and anatomical substrates of PPS can help advance our knowledge of the brain mechanisms underlying a wide range of neuropsychiatric conditions.

Over the past few decades, research focusing on the biological foundation of PPS has primarily relied on studying the physiology and anatomy of primates. This body of influential literature has identified a network of interconnected regions in the prefrontal, parietal, and subcortical areas, which exhibit sensory responses across multiple modalities. These regions have been suggested to form a circuit involved in integrating visual, tactile, and motor information (Rizzolatti et al. 1981, 1998; Gentilucci et al. 1988; Graziano and Gross 1993). Early in-vivo investigations in humans, based on task-based functional magnetic resonance imaging (fMRI), have largely supported these findings (Bremmer et al. 2001; Sereno and Huang 2006; Makin et al. 2007; Gentile et al. 2013; see di Pellegrino and Làdavas 2015 for a review). However, it has become evident that multisensory integration represents only a small piece of the puzzle, and the precise mechanisms underlying the representation of PPS within this circuit, as well as its interactions with other brain systems involved in sensorimotor, emotional, and social information processing, are still poorly understood.

In recent decades, advancements in the study of structural and functional connectomics have provided substantial evidence of distinct patterns of brain activity organization, with segregated, yet integrated modules that dynamically interact during rest and task-based activity (van den Heuvel et al. 2009; Eickhoff et al. 2011; Laird et al. 2013; Raichle 2015; Margulies et al. 2016; Basile et al. 2022a). The field of network neuroscience, which focuses on mapping, recording, analyzing, and modeling the interactions between elements of brain networks at multiple scales (Bassett and Sporns 2017), has introduced a fresh approach to investigating longstanding cognitive neuroscience questions.

The objective of this narrative review is to provide a comprehensive and up-to-date overview of the morphofunctional foundations of PPS processing in the human brain. Given the broad and inter-disciplinary nature of the topic treated, a narrative review approach was chosen. Papers were retrieved from online sources including PubMed, Scopus, EMBASE, Scholar, APA PsycNet, using terms related to PPS, neuroanatomy, neurophysiology, and neuroimaging. No specific constraints on the type of study, year of publication, and number of subjects were applied.

By synthesizing findings from various research methods, we will examine the anatomical aspects of the brain circuitry that underlie PPS representation and its interactions with higher-order brain functions. We will also explore the proposed PPS network and discuss recent insights from computational network neurosciences.

## The frontal lobe serves as a mediator for motor, cognitive, and social dimensions of PPS representation

Previous studies have consistently linked the processing of PPS to frontal cortical regions. The initial conceptualization of PPS as a distinct neural representation of the space within arm’s reach, stemmed from observations of visual-tactile neurons located in the posterior part of the monkey’s premotor cortex (Rizzolatti et al. 1981).

The premotor cortex (PMC), situated in the frontal lobe just anterior to the primary motor area (M1), is typically divided into dorsal (PMD) and ventral (PMV) portions. Within these regions, further subdivisions can be identified, resulting in four distinct areas characterized by their unique cytoarchitectonic and physiological features.

These areas include the dorsal caudal premotor cortex (PMDc or area F2 in the macaque monkey), which is involved in reaching movements; the ventral caudal premotor cortex (PMVc or F4), associated with sensorimotor integration; the dorsal rostral premotor cortex (PMDr or F7), responsible for learned responses to sensory stimuli; and the ventral rostral premotor cortex (PMVr or F5), specialized in grasping and hand-to-mouth movements (Matelli et al. 1985; Preuss et al. 1996; Rizzolatti et al. 1998). It is worth noting that the PMVr region also contains a specific class of visuo-motor neurons known as mirror neurons, which exhibit activity not only during the execution of movements but also when observing similar actions performed by others (Rizzolatti et al. 1996). Each of these regions contains motor representations of different body parts organized in a somatotopic manner (Godschalk et al. 1995). Of note, the neurons representing specific body parts are not confined to individual regions; instead, there are strong interconnections between neurons sharing the representation of the same part of the body. These connections exist within different areas of the premotor cortex, between the premotor and primary motor cortex (Muakkassa and Strick 1979; Barbas and Pandya 1987; Morecraft and van Hoesen 1993), and also between the premotor cortex and other lobes, such as the parietal lobe (Rizzolatti et al. 1998).

Neurons located in PMVc, which is close to the arcuate sulcus in monkeys, exhibit responses to tactile, visual, and auditory stimuli. When it comes to visual stimuli, these neurons prefer objects situated a few centimeters away from the skin (pericutaneous neurons) or within reach. Their receptive fields are not influenced by eye or body movements but rather appear to be anchored in a reference system centered on the head, trunk, and upper limb together (Rizzolatti et al. 1981; Gentilucci et al. 1988; Graziano 1999). For instance, visual receptive fields located in PMVc will respond to objects near the arm, whether the arm is stretched or held close to the body. These responses are independent of gaze shifts, indicating that the encoding of stimuli is not based on retinal position but on the position of the limb (Fogassi et al. 1992; Graziano et al. 1994). PMVc also exhibits a response to looming visual stimuli, which are perceived as approaching the face, and their activity is enhanced when coupled with congruent tactile stimulation (Guipponi et al. 2015; Cléry et al. 2017). Notably, microstimulation of PMVc elicits defensive movements such as shoulder shrugging, grimacing, and hand lifting, highlighting the role of PPS encoding in the premotor cortex in generating predefined behavioral responses to perceived threats (Graziano and Cooke 2006).

PMVr contains visuo-motor neurons that exhibit selective responses to stimuli in the PPS around the hand. Interestingly, these responses are not influenced by the distance or spatial properties of the stimuli, but rather by their operational properties, such as shape, size, and geometrical characteristics, which determine their “graspability” (Murata et al. 1997; Raos et al. 2006; Bonini et al. 2014). Mirror neurons in PMVr also display preferential activity for stimuli located in the PPS (Caggiano et al. 2009). Recent studies using fMRI in primates have revealed a weak preferential response for visual stimuli in the PPS in other areas like PMDv (F2), PMDr (F7), and the supplementary eye field (SEF) (Cléry et al. 2018).

In the human brain, fMRI investigations have produced similar findings, despite the challenges of identifying human equivalents of the primate premotor cortex. Rizzolatti and colleagues have proposed that, in humans, the homologous of monkey’s F4, extends within Brodmann area 6 (BA6) and BA44, located respectively in the rostral bank of the precentral gyrus and the most caudal bank of the inferior frontal gyrus, in proximity of the inferior frontal junction. The upper limits of the frontal eye field (FEF) are considered division landmarks between PMD and PMV in humans since the arcuate sulcus, present in primates, is absent in humans (Rizzolatti et al. 2002).

Most studies on human PPS have focused on multi-sensory stimuli delivered to the hand or the head. While early task-based fMRI investigations of the PPS around the hand reported activation of the IFG (Makin et al. 2007), other studies reported involvement of the rostral precentral sulcus, both in its dorsal and ventral parts (Brozzoli et al. 2011, 2012; Gentile et al. 2013; Limanowski and Blankenburg 2015). The human PMV is also responsive to auditory stimulation, being activated both by looming sounds alone (Seifritz et al. 2002), or when coupled with tactile stimulation to the hand (Ferri et al. 2015). Furthermore, a recent electrophysiological investigation employing intracranial electrodes suggests the involvement of the precentral gyrus in audio-tactile integration of looming auditory stimuli with tactile stimuli directed to the trunk (Bernasconi et al. 2018). Along with auditory, visual, and tactile integration, fMRI studies have yielded evidence supporting the integration of proprioceptive cues regarding the position of the arm and hand within the human PMV (Gentile et al. 2013). These findings suggest that the PMV combines multiple sensory inputs to form a comprehensive representation of the arm and its immediate environment (Limanowski and Blankenburg 2016). Additionally, investigations on visuo-tactile integration of looming stimuli directed to the head have reported event-related activity in the PMV and FEF (Huang et al. 2018).

Interestingly, the visual response in human PMV is stronger when another person’s hand is presented near the subject’s hand when compared to a prosthetic hand(Brozzoli et al. 2013).

This observation implies that PMV in humans may exhibit specific selectivity for socially relevant visual stimuli, such as the presence of other individuals. This aligns with previous studies indicating mirror-like neuronal activity in PMV and supports the hypothesis of mirror neurons’ presence in the human premotor cortex and their role in encoding the PPS (Buccino et al. 2001; Sakreida et al. 2005). Furthermore, inter-trial variability in PMV activation in response to looming audio-tactile stimuli reflects individual differences in the perceived boundaries of the PPS, suggesting that PMV encoding of PPS is not fixed but rather dynamic and subject to inter-individual variations (Ferri et al. 2015).

Moreover, activity in the human PMV is believed to contribute to the formation and maintenance of the sense of body ownership. The rubber hand paradigm, which involves concealing the real hand while synchronously stroking a visible prosthetic hand, has been employed to manipulate the sense of hand ownership and location (Botvinick and Cohen 1998). Task-based fMRI experiments utilizing this paradigm have demonstrated that premotor cortex activity correlates with the sense of ownership of the prosthetic hand(Ehrsson et al. 2004). Additionally, during this illusion of limb ownership, PPS-related response in the PMV is consistently shifted towards stimuli located near the prosthetic hand, and the strength of such response correlates with the perceived sense of ownership (Brozzoli et al. 2012). By generalizing the rubber hand illusion paradigm to a whole-body perceptual illusion, Gentile and colleagues demonstrated a similar relationship between PPS encoding and perceived whole-body ownership in the PMV, along with identifying regions of the PMV encoding for PPS around specific body parts (Gentile et al. 2015).

Taken together, these results suggest that, in line with primate studies (Cléry et al. 2015), human premotor regions such as PMD, PMV, and FEF encode multisensory stimuli located in the PPS and may be relevant to translate information located around the head or hand into appropriate motor outputs (Rizzolatti et al. 1998, 2002). Moreover, human neuroimaging studies indicate that PMV activity is sensitive to higher-order psychological factors such as the perceived ownership of the hand and socially relevant information, like the presence of others in the PPS. This highlights PMV as a pivotal structure connecting motor, cognitive, and social functions of PPS representation.

## Perceptual, cognitive, and attentional aspects of PPS are mediated by parietal areas

In monkeys, the parietal areas first described as involved in the coding of PPS were area 7 and the intraparietal areas (Rizzolatti et al. 1998; di Pellegrino and Làdavas 2015; Cléry et al. 2015). Area 7 in monkeys is further subdivided into areas 7a and 7b, extending both in the lateral and medial hemispheric surface of the inferior parietal lobule (Andersen 1989). In humans, these areas are located in the superior parietal lobule. The intraparietal areas, including the anterior portion (AIP), lateral portion (LIP), and ventral portion (VIP), are situated within the depths of the lateral aspect of the intraparietal sulcus (Andersen and Buneo 2002). In analogy to premotor cortices of the frontal lobe, these regions exhibit functional specialization in visuospatial processing related to motor planning and execution, forming parietal-premotor circuits with strong connectivity to premotor areas of similar functional specialization (Rizzolatti et al. 1998; Cléry et al. 2015). Areas 7b and AIP contain visuotactile neurons that respond to both simple and complex movements, such as grasping (Hyvärinen and Shelepin 1979; Hyva¨rinen 1981), as well as mirror neurons that respond to the visual observation of hand actions (Fogassi and Luppino 2005; Maeda et al. 2015). On the other hand, AIP does not contain visuotactile neurons but instead comprises visual-dominant, visuo-motor, and motor-dominant neurons that discharge during grasping actions based on lighting and object visibility (Sakata and Taira 1994). The AIP-7b-F5 pathway is thought to integrate visual and tactile information for grasping and tool use, contributing to the representation of the PPS around the hand (Rizzolatti et al. 1998, 2002; Cléry et al. 2015). Additionally, the visual receptive fields of AIP are known to expand after repetitive tool use, suggesting its involvement in adaptive processes contributing to the reshaping of PPS followed by object manipulations(Maravita and Iriki 2004).

LIP is a prominently visual area involved in the planning of eye movements, possesses a retinotopically organized map of saccadic movements, and has strong interconnections with FEF and SEF (Ben Hamed et al. 2001; Janssen et al. 2008; Cléry et al. 2015). Neuronal responses in the LIP are biased towards stimuli in the PPS (Gnadt and Mays 1995; Genovesio and Ferraina 2004), and its visual receptive fields are modulated by attentive fixation (Ben Hamed 2002). Taken together these results suggest that the LIP-FEF-SEF circuitry may have a role in enhancing visuospatial attention towards stimuli in the PPS and guiding ocular motion accordingly.

Finally, VIP shares functional similarities with premotor area F4 (Rizzolatti et al. 1998), receives inputs from multiple sensory modalities, including visual, tactile, and auditory sources (Duhamel et al. 1998; Schlack et al. 2005; Chen et al. 2014), and integrate visuotactile stimuli (Guipponi et al. 2015; Cléry et al. 2018). Compared to F4, VIP receives also prominent vestibular input, suggesting that it integrates relevant information from head motion, and, in general, ego-motion in space (Chen et al. 2011a, b, 2013). This is probably related to another relevant difference between PPS encoding in VIP and F4. While the former represents space mostly in arm-centered coordinates, PPS representation in VIP is anchored to the head and only partly independent from gaze direction (Avillac et al. 2005). Interestingly, some neurons in VIP which respond to the visual presentation of objects in the proximity of a certain body part, also respond when the same object is in proximity of the same part of the body of the experimenter (“body matching neurons”) (Ishida et al. 2010). Overall, these findings suggest a role for the VIP-F4 circuit in mediating social aspects of PPS processing.

In humans, many neuroimaging studies have strived to identify homologous parietal regions involved in PPS processing. Task-based fMRI investigation has identified a homologous region of primate AIP, which is activated by visually guided grasping (Shikata et al. 2003; Culham et al. 2003), but also in observation and imitation of movements (Mühlau et al. 2005), and observation of graspable objects (Chao and Martin 2000). In line with research in the non-human primate, this region is also activated in fMRI paradigms focused on the PPS around the hand (Makin et al. 2007; Brozzoli et al. 2011, 2012, 2013; Gentile et al. 2013), enforcing the idea of a tight link between peri-hand PPS and planning of appropriate grasping movements.

A human homologous of monkey’s LIP has been identified by using retinotopic mapping and has been implicated in gaze-centered updating of visual space (Sereno et al. 2001; Medendorp et al. 2003), as well as in response to looming visuo-tactile stimuli (Huang et al. 2018).

In the case of VIP, evidence of multimodal visual, tactile, and auditory integration in the proximity of the IPS has been provided through event-related fMRI (Bremmer et al. 2001). Using surface-based fMRI, researchers have described a detailed map of the human face that integrates visual and tactile inputs (Sereno and Huang 2006; Huang et al. 2012). Additionally, high-resolution fMRI with retinotopy mapping demonstrated the alignment of retinotopic and tactile maps; however, such parietal face map is located more anteriorly and ventrally compared to its counterpart in monkeys, which occupies the fundus of the IPS (Huang et al. 2017). Further, multiple fMRI studies suggest that other functions associated with VIP in non-human primates, such as optic flow movement (Konen and Kastner 2008; Sulpizio et al. 2020), or ego-motion and vestibular conscious perception (Schindler and Bartels 2018; Aedo-Jury et al. 2020), involve different regions in the IPS and the surrounding parietal cortex. It has been proposed that, in the human brain, VIP may have evolved into a diffuse VIP complex, involving different parietal regions responsible for the representation of the head in space, visual head direction, and coding of the PPS around the head (Foster et al. 2022).

Along with areas traditionally involved with the encoding of PPS-related information, other parietal regions have been suggested to play a role in PPS representation in the human brain.

A recent meta-analysis of neuroimaging studies has reported the activation of both postcentral gyrus (PoCG), SPL (BA5, BA7), and IPL (BA 39, 40) (Grivaz et al. 2017). PoCG was the region where the most prominent PPS effect was observed in an intracranial EEG study aimed at investigating temporal dynamics of audio-tactile integration in the trunk-centered coordinate system (Bernasconi et al. 2018). Although traditionally conceived as subserving unimodal processing, some evidence suggests that PoCG may be involved in multisensory integration. As an example, the vision of a body part being touched is known to pre-activate the corresponding somatotopic region in PoCG, thus influencing tactile sensation (Taylor-Clarke et al. 2002; Blakemore et al. 2005; Longo et al. 2011; Schaefer et al. 2012).

SPL includes BA5 and BA7, which are known to subserve integration of tactile and visual stimuli, and it has been involved in visuospatial attention tasks (Macaluso and Driver 2005; Wu et al. 2016). An area putatively labelled as 7b has shown activation for approaching visuotactile stimuli directed to the face (Huang et al. 2018). This is in line with evidence in primates and suggests that, in the human brain, SPL could be involved in driving visuospatial attention towards stimuli intruding on the PPS.

The inferior parietal lobule (IPL) is commonly described as consisting of the supramarginal gyrus and angular gyrus (respectively BA39 and BA40); the supramarginal gyrus has been identified as a site of multisensory integration when visuo-tactile stimuli were presented in the peri-hand space (Gentile et al. 2011; Brozzoli et al. 2012).

The parietal operculum was also identified as a site of multi-sensory processing of information from the hand (Gentile et al. 2011); this region has been subdivided into four cytoarchitectonic areas (OP1-4), which have been described as a secondary somatosensory area (SII) (Disbrow et al. 2000). Joint anatomical and functional MRI evidence suggests that OP4 is densely connected with frontal premotor regions and may subserve aspects of sensorimotor integration (Eickhoff et al. 2010). Finally, posterior sylvian regions including SII have also shown intense activation for looming visual and tactile stimuli and visual-auditory stimuli in event-related fMRI investigations (Tyll et al. 2013; Huang et al. 2018).

To summarize, a wide range of parietal regions play distinct roles in various processes related to PPS representation. While certain areas in the parietal lobe exhibit strong connections with functionally similar regions in the premotor cortex, their primary involvement lies in the integration of multiple sensory modalities for PPS representation rather than encoding specific behavioral responses. Moreover, while both frontal and parietal representations of PPS are relative to the whole body, frontal representations specifically emphasize PPS around the upper limb and arm, while parietal representations are more focused on PPS around the head and face. A close relationship between visuospatial attention, regulation of ocular and head movements, and encoding of PPS becomes evident when considering the anatomical and functional organization of the parietal nodes within the PPS network. Hence, while frontal representations of PPS appear to contribute to motor planning, defensive responses, and social aspects, parietal regions are mostly involved in the perceptual and cognitive/attentional aspects of PPS processing (Table 1).

**Table 1:** Similarities and differences between PPS representation in the frontal and parietal lobes. The table summarizes the main elements of PPS representation in terms of the type of spatial representation, sensory input and motor output channels, and behavioral relevance. Despite being considered here as separate entities, it should be kept in mind that these regions are part of strictly interconnected integrative circuits. *PMV: ventral premotor cortex; IPS: inferior parietal sulcus*

Features of PPS representation	Frontal lobe (PMV)	Parietal lobe (IPS)
Spatial representation	Centered on specific body segments	Centered mostly on the head
Sensory channels	Visual, tactile, auditory, proprioceptive	Visual, tactile, auditory, vestibular
Motor output channels	Arm movements	Eye movements
Behavioral relevance	Grasping, reaching, defensive behavior	Visuospatial attention

## Interactions between fronto-parietal and distributed cortical regions to shape specific features of PPS

As discussed in the previous paragraph, the study of PPS representation has predominantly relied on primate research using in-vivo electrophysiological techniques. These methods have successfully revealed a closely interconnected system of frontal and parietal regions involved in multisensory PPS representation, and these findings have been largely supported by human neuroimaging studies. However, beyond the canonical areas associated with PPS processing, functional investigations in humans have provided evidence for the involvement of other cortical regions in specific aspects of PPS representation.

In this regard, while most of the research investigating PPS processing in human and non-human primates has focused on visuo-tactile integration, auditory paradigms based on looming sounds or looming audio-visual and audio-tactile stimulation have also suggested the involvement of temporal lobe regions in the auditory PPS representation (Tyll et al. 2013; Ferri et al. 2015; Bernasconi et al. 2018). Notably, lower-level auditory regions such as the auditory belt regions located in the *planum temporale* (PT) exhibit a preference for rising sounds, which correspond to sounds approaching the boundaries of PPS (Warren et al. 2002; Seifritz et al. 2002; Warren and Griffiths 2003; Krumbholz et al. 2005). The superior temporal sulcus (STS), along with being activated by auditory looming stimuli (Seifritz et al. 2002; Hall and Moore 2003; Bach et al. 2009), is involved in multi-sensory integration, as it contains visual neurons responding to the sight of movement (Beauchamp et al. 2004; Barraclough et al. 2005; Huang et al. 2018). Furthermore, audio-visual, and audio-tactile congruent stimulation elicited activity in the STS (Tyll et al. 2013; Ferri et al. 2015). Taken together, these data suggest that temporal lobe regions may be relevant in PPS representation by binding auditory-related information with visual approaching stimuli, contributing to the prediction of tactile impact on the body.

Along with auditory-related temporal lobe regions, visual areas of the occipital lobe have been also involved in PPS representation.

For instance, the parieto-occipital junction (POJ), the homologous of monkey area V6 (Pitzalis et al. 2013), has demonstrated a preference for near vs. far viewing in different fMRI investigations (Quinlan and Culham 2007; Wang et al. 2016; Huang et al. 2018). Recent evidence suggests that POJ acts as an interface structure between the so-called dorsal and ventral visual streams, the first being involved in visuospatial processing and the latter in object identification (Goodale and Milner 1992; Shmuelof and Zohary 2005). It may be hypothesized that stimuli in the PPS, which are immediately recognized as relevant for action, elicit a preferential involvement of the dorsal stream, while stimuli in far space would preferentially drive ventral stream activation for object recognition (Previc 1998; Gilaie-Dotan 2016): in this framework, POJ would act as a switch by integrating information of dorsal and ventral stream areas in response to far vs. near stimuli (Wang et al. 2016).

A possible role in PPS representation has been also suggested for the lateral occipital cortex (LOC), specifically for a region that is known to respond to body-related visual stimuli, the so-called extrastriate body area (EBA) (Downing et al. 2001). This region is co-activated, together with PMV and inferior parietal regions, during bodily illusions obtained using congruent visuo-tactile stimulation (Gentile et al. 2011, 2013; Sonobe et al. 2023) and is thought to play a role in mediating the sense of body ownership (Grivaz et al. 2017). It has been proposed that EBA mediates the integration of other subjects and their PPS into one’s PPS representation, thus mediating the interpersonal, social modification occurring in the PPS in the presence of conspecific subjects (Fanghella et al. 2021).

Finally, the contribution of limbic regions to PPS representation has also been proposed to mediate the interaction between PPS representation and higher-order cognitive and emotional functions. The anterior cingulate cortex (ACC), located in the rostral part of the cingulate gyrus, has been involved in multiple functions including context-dependent multimodal sensory and motor integration, error detection, and social functions (Paus 2001; Apps et al. 2016). Although not directly associated with PPS representation, several studies have reported the activation of ACC in response to stimuli within the PPS. Both in primates and human subjects, ACC activation has been observed during synchronized tactile and visual stimulation of the face and upper limbs (Cléry et al. 2017; Huang et al. 2018). It is worth noting that tactile stimulation of the face, particularly in non-human primates, is perceived as a stressful and aversive stimulus. Among its many possible functional roles, evidence links ACC activity to threat detection and anticipation (Singer et al. 2004; Morrison et al. 2004; Andrzejewski et al. 2019). In line with this hypothesis, fMRI studies have demonstrated ACC activation when subjects are exposed to looming threatening stimuli, such as virtual predators, tarantulas, or threatening avatars (Mobbs et al. 2007, 2010; Vieira et al. 2020; de Borst and de Gelder 2022).

The anterior insular cortex (AIC) has also been implicated in PPS representation (Huang et al. 2018; Bernasconi et al. 2018). The insula is a well-known center for multisensory integration, and activation of the AIC has been associated with the timing and synchrony of convergent sensory stimuli (Bushara et al. 2001, 2003; Calvert et al. 2001; Cappe et al. 2012). Additionally, similar to the ACC, the AIC is involved in attributing perceptual salience to emotionally relevant or noxious stimuli (Singer et al. 2004; Jackson et al. 2005; Wiech et al. 2010). In this context, activation of AIC has also been observed during the perception of approaching emotionally salient stimuli such as hostile faces or avatars (Lloyd et al. 2006; Vieira et al. 2017; Schienle et al. 2017; de Borst and de Gelder 2022).

The ACC and AIC could also be involved in mediating bidirectional interactions between PPS representation and nociception. These brain regions are known to play a pivotal role in both physical and affective pain perception (Medford and Critchley 2010; Xiao and Zhang 2018). In analogy to other sensory stimuli, it has been demonstrated that noxious stimuli are mapped on a spatial representation that is anchored to the PPS (Haggard et al. 2013; De Paepe et al. 2014). Accordingly, the ability to perceive nociceptive stimuli on the skin is influenced by the integration of visual stimuli presented near the body space (De Paepe et al. 2017; Filbrich et al. 2017a). In addition, following experimentally induced embodiment of a virtual body, a network involving the PMV and ACC showed time-locked decreased activity in response to pain-related vicarious somatosensation compared to touch-related vicarious somatosensation (Pamplona et al. 2022). Since ACC and AIC are also involved in affective pain-related processes such as empathic pain (Timmers et al. 2018), bidirectional interactions between areas involved in PPS representation and these regions could mediate the enhancing effect of physical and psychological closeness on empathic pain perception (Grynberg and Konrath 2020).

Taken together, these findings suggest that specific aspects of PPS representation may be mediated by the interaction between fronto-parietal areas and a wide set of distributed cortical regions.

## Subcortical contributions to PPS representation

Although often overlooked in research regarding the neural substrates of human PPS representation, many subcortical regions are known to contribute to different features of PPS encoding.

Structures of the basal ganglia and the cerebellum are part of a complex circuitry involved, among other functions, in sensorimotor integration (Milardi et al. 2019; Basile et al. 2022b), and they may take part in integrative processes related to PPS processing.

Putamen, a key structure in basal ganglia circuitry, has been implicated in the neural mechanisms underlying PPS since early investigations in the macaque brain, where visuo-tactile neurons with properties similar to those observed in the PMVc, area 7b, and VIP were discovered (Graziano and Gross 1993). The putamen contains a somatotopically organized map of the human body (Nambu 2011), with visuotactile neurons located near the hand and head regions. The visual receptive fields of these neurons are associated with representations of the head or upper limbs(Graziano and Gross 1993). As part of the dorsal striatum, the putamen receives topographically organized connections from various cortical regions (Cacciola et al. 2017; Milardi et al. 2019; Bertino et al. 2020; Quartarone et al. 2020; Basile et al. 2021), including the PMV and intraparietal areas; the territories of innervation of these two regions show strong convergence (Gerbella et al. 2016), in line with the observation that strongly interconnected regions of the cerebral cortex generally show overlapping striatal territories (Shipp 2017). Human fMRI studies focused on the PPS around the hand have demonstrated co-activation of the putamen with PMV and IPS, suggesting that the PMV-IPS-putamen circuitry may mediate multisensory representations of PPS around the hand (Gentile et al. 2011; Brozzoli et al. 2011) However, further research is needed to shed light on the precise contribution of the putamen in the computational processes involved in PPS representation.

The cerebellar lobule VI has also been found to be involved in multisensory integration when stimuli were presented in the peri-hand space (Gentile et al. 2011; Brozzoli et al. 2011). Cerebellar lobule VI is part of one of the two somatotopically organized representations of the human body in the cerebellum, specifically hosting a representation of the head and hand (Grodd et al. 2001; Guell et al. 2018; Boillat et al. 2020). In addition, cerebellar lobule VI has been shown to respond to visual and auditory moving stimuli (Baumann and Mattingley 2010).

While traditionally considered as primarily involved in motor functions, emerging research suggests that the cerebellum may also contribute to higher-order functions, including perception (Baumann et al. 2015). The basilar pontine nuclei, which provide cortical input to the cerebellar cortex, exhibit overlapping projections from the premotor cortex and parietal sulcus regions, suggesting a potential role in convergence processes (Schmahmann and Pandya 1989, 1997; Schmahmann 1996).

Therefore, it is plausible that the cerebellum is involved in multisensory integration but the extent to which these processes are related to PPS representation requires further investigation.

In addition to multisensory and sensorimotor integration, subcortical regions implicated in visuospatial attention and the detection of visually salient stimuli may also contribute to PPS-related circuitry.

The pulvinar, a higher-order thalamic nucleus, exhibits diverse connections with visual areas involved in the ventral and dorsal streams (Benarroch 2015). Specifically, the medial nucleus of the pulvinar shows reciprocal connectivity with temporal and parietal areas involved in the processing of moving stimuli (Burton and Jones 1976; Baleydier and Morel 1992). In line with these findings, preferential activation in response to looming stimuli has been observed in the pulvinar, and putatively located in the medial pulvinar (Billington et al. 2011; Huang et al. 2018). Moreover, the medial pulvinar has been proposed to mediate emotionally based attention mechanisms due to its connection with the superior colliculus (SC) and the amygdala (Jones and Burton 1976; Saalmann et al. 2012; McFadyen et al. 2019).

In this regard, the SC is a phylogenetically older structure involved in visual processing. Located in the tectal region of the midbrain, it is known to be involved in the detection of visually salient stimuli to drive oculomotor responses (Wurtz and Albano 1980; Gandhi and Katnani 2011; White et al. 2017). In line with this role, SC also has been shown to respond to looming visual stimuli, which have to be recognized as immediately salient for the individual (Billington et al. 2011; Vieira et al. 2020).

On the other hand, the amygdala is involved in stimulus-value association, recognition of emotionally salient stimuli, and orchestration of an appropriate behavioral response (Phelps and LeDoux 2005; Paton et al. 2006; Baxter and Croxson 2012). Functional MRI studies in humans have revealed preferential activation of the amygdala for approaching, rather than static or receding, emotionally salient stimuli such as faces (Holt et al. 2014; Wabnegger et al. 2016; Vieira et al. 2017; Schienle et al. 2017).

Consistent with the “fast route” hypothesis, which postulates that the SC-pulvinar-amygdala pathway may facilitate rapid adaptive response to emotionally relevant stimuli (McFadyen et al. 2019), it is tempting to hypothesize that reciprocal cortical connections from PPS-relevant areas may integrate into this circuit relevant multi-sensory information about PPS in both a bottom-up and top-down fashion.

Supporting this hypothesis, a bias towards looming and approaching stimuli has been also observed in structures directly involved in orchestrating the behavioral response to pain and threatening stimuli, such as the periaqueductal grey (Mobbs et al. 2007; Coker-Appiah et al. 2013; Vieira et al. 2020).

To summarize, the convergence of cortical inputs in the cerebellum and the reciprocal connections between the pulvinar, superior colliculus, and amygdala suggests that these subcortical structures may integrate PPS-related information in a dynamic and contextually relevant manner. These subcortical circuits contribute to multisensory integration, sensorimotor processing, visuospatial attention, and the detection of salient stimuli within PPS. However, further research is needed to unravel the precise mechanisms and computational processes through which these subcortical regions contribute to PPS representation, shedding light on the complex neural underpinnings of our perception and interaction with the space immediately surrounding our bodies.

## PPS encoding in a network perspective: core and extended PPS subsystems and their relation to intrinsic connectivity networks

As pointed out in the previous paragraphs, PPS representation in the human brain is the result of complex and highly faceted neural computational processes occurring in different brain regions and involving the integration of sensory, motor, attentional, cognitive, emotional, and social information. While the locationist theory of the brain focused on the investigation of specific functions within brain regions, the last decade has seen a paradigmatic shift toward a network-based approach, setting the focus on how distinct brain regions are interconnected together and interact in space and time from a structural and functional point of view (Sporns 2011; Uddin 2013). The concept of brain network, although variously defined in the literature, always refers to a distinct and discrete set of brain areas that are strictly interconnected together, forming segregated, yet mutually interacting modules at different hierarchical levels (Bullmore and Sporns 2009; Uddin et al. 2019). While in earlier research the term “network” is generically applied to interconnected areas showing evidence of cooperative activity toward specific brain function (hypothesis-driven) (Mesulam 1990), a core concept of modern network neuroscience is that network properties can be inferred through data-driven analysis of large-scale connectivity data (Bullmore and Sporns 2009; Buckner et al. 2009; van Wijk et al. 2010; Yeo et al. 2011). Aimed at translating these concepts to the field of PPS research, and to the investigation of its functional and anatomical correlates, a putative PPS network could be identified by gathering information on brain regions involved in PPS representation.

As pointed out in the previous sections, premotor and parietal regions involved in the PPS show evidence of strong reciprocal interconnections. Functionally homologous regions in the frontal and parietal lobe are tightly interconnected to each other forming circuits that underlie specific aspects of PPS representation: (i) a VIP-PMVc circuit for the space around the arm and head to subserve defensive behavior; (ii) a 7b-AIP-PMVr circuit for grasping; (iii) and a LIP-FEF circuit for ocular motion and attention (Rizzolatti et al. 1998; di Pellegrino and Làdavas 2015; Cléry et al. 2015). Together, these fronto-parietal circuits can be regarded as the core of the PPS network as they are involved in the basic multisensory integration processes and action representation needed to adequately process information from the near space and generate appropriate behavioral responses.

**Fig. 2:**
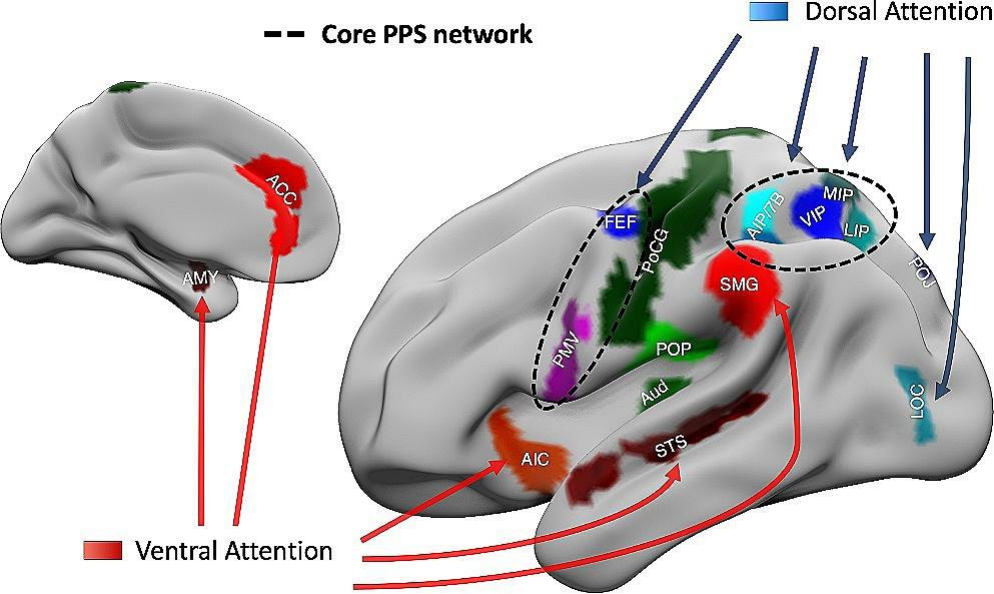
Core and extended PPS networks. A graphical representation of brain circuits involved in PPS representation. Areas that are part of the core PPS network are circled with dashed lines. Regions are classified according to their relevance to large-scale brain networks, highlighted by different colors (blue: dorsal attention network; red: ventral attention network; green: sensorimotor network). Cortical labels are obtained from the HCP multimodal parcellation atlas (HCP-MMP). ACC: anterior cingulate cortex; AIC: anterior insular cortex; AIP: anterior intraparietal; Aud: auditory belt; FEF: frontal eye field; LOC: lateral occipital cortex; PMVc: caudal ventral premotor; PMVr: rostral ventral premotor; PoCG: postcentral gyrus; POJ: parieto-occipital junction; POP: parietal operculum; SMG: supramarginal gyrus; STS: superior temporal sulcus

However, neuroimaging studies have indicated that a broader set of cortical and subcortical areas may interact with the core PPS network. These additional regions encode diverse information related to one’s own body or the body of others, as well as their spatial position (such as PoCG, SII, LOC, or cerebellar lobule VI) (lriki et al. 1996; Disbrow et al. 2000; Grodd et al. 2001; Eickhoff et al. 2010; Bernasconi et al. 2018; Sonobe et al. 2023), visual and auditory space representation and movement detection (such as PT, STS, POS, or at a subcortical level, SC, pulvinar and the cerebellum) (Seifritz et al. 2002; Baumann and Mattingley 2010; Billington et al. 2011; Wang et al. 2016), emotional and behavioral relevance of stimuli (such as ACC, AIC, amygdala or PAG) (Mobbs et al. 2010; Vieira et al. 2020; de Borst and de Gelder 2022). Such an extended PPS network may work in concert with the core PPS network to integrate information from other perceptual domains in the PPS representation and, possibly, to filter and update lower-level sensory processing in light of the existing operational models of PPS (Fig. 2).

Support for this hypothesis can be found in intrinsic connectivity networks derived from either task-based fMRI co-activation or resting-state functional connectivity analysis. A network including the IPS and surrounding areas and involving primarily the FEF has been identified as the *dorsal frontoparietal network* or *dorsal attention network* (Corbetta and Shulman 2002; Szczepanski et al. 2013); this network may also expand to include the PMV or corresponding areas, such as the inferior frontal junction (Toro et al. 2008; Corbetta and Shulman 2011; Gordon et al. 2017). It has been theorized that nodes of this network may cooperate into building a multi-modal, feature-independent priority map of the environment, by coupling sensory and spatial information about stimuli with behavioral planning and goal representations encoded in prefrontal and premotor regions (“goal-directed attention”) (Corbetta and Shulman 2002; Ptak 2012). In this context, considering the intrinsic relevance that stimuli located in the surroundings of the body have for goal-directed attention and motor planning, the involvement of core regions involved in PPS representation is not surprising.

On the other hand, regions involved in the detection of behaviourally relevant environmental stimuli have been often identified as part of the so-called *salience network*. Core nodes of this network include the anterior cingulate cortex, the anterior insula, the superior temporal gyrus, and the amygdala (Seeley et al. 2007; Yeo et al. 2011; Gordon et al. 2017; Seeley 2019), though the involvement of supramarginal gyrus and premotor cortices have been also reported (Fox et al. 2006; Corbetta and Shulman 2011). This network has been suggested to mediate the shifting of attention towards internal or external salient stimuli (“stimulus-driven attention”) and to broadly overlap with the *ventral attention network* (Corbetta and Shulman 2002; Vossel et al. 2014). Additionally, it should be noted that, together with the cingulo-opercular network, the ventral attention and salience networks, are encapsulated in the midcingulo-insular salience network. As previously described, some of these regions show biased activity towards stimuli in the PPS or activate in the presence of behaviourally relevant stimuli approaching the PPS. The involvement of some regions, such as the ventral premotor cortices or the inferior frontal junctions, both in the dorsal and ventral attention network should not surprise as the extent and nodal definitions of brain networks can vary widely between different studies (Uddin et al. 2019). In addition, dynamic interaction between brain networks has been pointed out as crucial for higher-order cognitive processing. Generalizing this concept, functional networks in the brain may not exist as static entities, but rather as superordinate approximations of dynamic, context-dependent states (Ciric et al. 2017).

The dorsal and ventral attention networks have been shown to co-activate during reorienting of visuospatial attention (Corbetta and Shulman 2011), and the ventral frontal cortical regions, including the PMV, may mediate the interaction between the systems (Vossel et al. 2014). In this regard, the functional connectivity pattern of the inferior frontal junction, which shows strong functional coupling with the frontoparietal network, shifts towards ventral regions during stimulus-driven attentional conditions (Tamber-Rosenau et al. 2018).

In summary, evidence from data-driven connectivity studies allows to collocate PPS-related brain regions in the wider picture of goal-directed and stimulus-driven attentional systems. Interestingly, areas involved in the multisensory transformations, necessary for creating a body-centered space representation, are part of a dorsal network involved in the spatial prioritization of objects, emphasizing the role of PPS in goal-directed spatial representation. On the other hand, brain regions encoding specific features of objects in the PPS, such as their movement towards the body or their emotional, social, or behavioral relevance are included in a wider ventral network involved in salience attribution. Lying at the watershed between these two systems, the ventral premotor cortex may represent a crucial node for their interaction, by ensuring the appropriate crosstalk between goal-directed and stimulus-driven aspects of PPS processing.

## PPS in pathological conditions

As highlighted previously, the perception of the space around one’s body is a fundamental mechanism that allows us to efficiently navigate and interact with the environment through a seamless integration between our sensory inputs and motor responses. In recent years, research has focused on understanding whether and how alterations in PPS perception are expressed in pathological conditions. Indeed, disturbances in PPS representation have been increasingly documented in various neuropsychiatric disorders, such as stroke, hemispatial neglect, schizophrenia, and autism spectrum disorder (ASD), and in traumatic injuries affecting the spinal cord and the peripheral nervous system. In this wide range of conditions, PPS disturbances have been associated with deficits in movement planning, body ownership, spatial awareness, and social interactions, thus underlying the paramount importance of PPS in maintaining motor control, spatial awareness, and adaptive social behaviors.

In this section, we delve into some conditions that display PPS alterations, including spinal cord injuries (SCI), phantom limb phenomena (PLP), stroke, and psychiatric disorders. Through this exploration, we aim to contribute to a deeper understanding of the intricate relationship between PPS perception, sensorimotor functioning, and our ability to efficiently interact with the environment, possibly informing innovative research and therapeutic strategies to address the symptoms (Table 2).

**Table 2:** PPS abnormalities in neurological and neuropsychiatric condition: hypotheses and perspectives

Neuropsychiatric condition	PPS alteration	Putative neuroanatomical substrate	Proposed treatment strategy
Spinal Cord Injury	Reduced PPS representation around the body parts	Interruption of sensory afferents to PPS network. Compensatory role of the anterior insula	PPS representation could be enlarged by passive manipulation of the affected limbs. Interoceptive awareness could enhance quality of PPS representation
Amputation and Phantom Limb Phenomena	Preserved PPS representation around the missing limb may contribute to the insurgence of PLP	Persistence of the neural circuitry underlying PPS representation of the missing limb	The maintenance of the pre-existing body schema and PPS borders can be used to boost the embodiment of a prosthesis
Hemispatial neglect	Dissociation of neglect severity between peripersonal and extrapersonal space	Lesion of posterior parietal cortex	PPS assessment may be included into the evaluation of neglect patients
Anxiety disorders	Expanded PPS may lead to diminished perception of “security boundaries” around the body	Hyperactivity in fronto-parietal networks underlying PPS representation	Virtual reality treatments for anxiety act by manipulating perception of the PPS
Schizophrenia	Reduced and blurred PPS boundaries correlate with negative symptoms and discriminate early onset vs. adult-onset schizophrenia	Unknown (altered excitatory-inhibitory imbalance?)	Multisensory integration through virtual reality could help reestablish normal PPS boundaries and improve social interactions
Autism Spectrum Disorder	Reduced, sharper PPS boundaries	Delayed multisensory integration	PPS representation may be improved using behavioral therapy for ASD

.

### Spinal cord injury

PPS representation is generated through the multimodal integration of sensorimotor inputs, which allows a dynamic and adaptive perception of the space in which actions can be performed. As such, PPS representation can also be affected even when the disruption of sensorimotor information originates from outside the cortical and subcortical brain regions, as in acquired spinal cord injuries (SCIs). Besides resulting in variable sensory and motor function impairments below the level of injury, recent evidence suggests that SCIs trigger adaptive changes in PPS perception, thus affecting the individual’s ability to interact with the environment. Scandola and colleagues designed a cross-modal integration paradigm (i.e., tactile and visual) to assess PPS representation around the legs in 18 paraplegic patients (Scandola et al. 2020), showing that SCIs are associated with reduced PPS representation around the body parts below the lesion level. Interestingly though, the shrinkage of PPS representation around the feet could be restored with 15 min of passive leg mobilization. Several factors could contribute to the restoration of PPS representation of paralyzed limbs with passive mobilization, including the presence of motor or visual feedback or interoceptive processes. A later study by the same authors (Scandola et al. 2020) demonstrated that, in paraplegic patients with both complete and incomplete lesions, passive movements were able to restore PPS representation independently from the presence of visual feedback. Importantly, patients with complete lesions were able to correctly identify whether their limb was moving even without visual feedback, thus suggesting that movement perception is partially spared even after a complete SCI. This remarkable finding supports the increasingly recognized role of interoception in the recovery of PPS, as corroborated by a positive relationship between the quality of the PPS representation and the level of interoceptive awareness (Scandola et al. 2020). It is worthy to note that AIC, that plays a crucial role in mediating interoceptive awareness (Wang et al. 2019), is part of the ventral attention network (Vossel et al. 2014) and has been implied in PPS representation (Lloyd et al. 2006; de Borst and de Gelder 2022). It would be then hypothesized that interoceptive information may compensate from loss of external sensory information, as occurring in peripheral sensory loss following SCI, and that this compensatory mechanism may be mediated by connections of the anterior insula (a part of the extended PPS network, as underlined in paragraph 5) with core regions involved in PPS representation; however, to our knowledge, direct evidence supporting this hypothesis is still lacking. Altogether these findings indicate that, while congruent multisensory information is fundamental for building accurate PPS representation in healthy individuals, sensory afferents and motor efferents play an important role in creating and reshaping PPS after SCI. In line with this view, restoring the impaired PPS representation after SCI, e.g. by motor imagery-based rehabilitation, has also been proposed as a possible treatment strategy, to ultimately improve sensorimotor deficits in patients suffering from these conditions (Moro et al. 2021). Accordingly, these studies contribute to the notion of embodied PPS, which takes into account the continuous dialogue between the environment and the body in shaping and adapting PPS representations.

### Amputation and Phantom Limb Phenomena

The concept of embodied PPS has important implications also for individuals whose body schema and PPS representation are altered following an amputation. Indeed, patients with upper limb amputations display a reduced PPS representation around the stump but, remarkably, wearable prosthesis malleably extends the PPS boundaries to include the prosthetic device (Canzoneri et al. 2013). Research on prosthesis embodiment – the process of perceiving and integrating a prosthesis as a natural extension of the body – has underscored the significance of PPS plasticity in promoting the integration and acceptance of prosthetic devices (Di Pino et al. 2020; Bekrater-Bodmann 2022). Notably, the use of prostheses has been associated with structural changes in the posterior parietal cortex, a pivotal region for multisensory integration, spatial and sensorimotor processing, and eye-hand coordination. Conversely, it has been negatively correlated with the experience of phantom limb sensations (Preißler et al. 2013). Phantom Limb Phenomena (PLP) reflect a false embodiment experience where individuals with an amputation or congenital missing limbs experience a wide range of sensations or even pain in the missing body part. Defined as a “continuous awareness of a non-existing or deafferented body part with specific form, weight, or range of motion (Ribbers et al. 1989), PLP is often reinforced by both non-conscious and intentional execution of actions involving the missing limb, even when such movement is observed (Giummarra et al. 2010). This suggests that the sensorimotor pathways connecting the peripheral nerves to their cortical areas are still preserved to some degree, as demonstrated by evidence of retained sensorimotor function for years after an amputation (Dhillon et al. 2004). Although unequivocal evidence is missing, this may also imply that PPS representations may be also preserved in amputees and contribute to the onset of PLP. Studies on phantom-like sensations in healthy individuals, typically elicited with the rubber hand illusion (Botvinick and Cohen 1998), demonstrated the intricate relationship between our sense of embodiment and PPS perception. For example, it has been demonstrated that healthy participants subject to the rubber hand illusion with one missing finger shared several sensory symptoms as patients with PLP, including feeling the finger that is not present, changes in size and shape of the missing finger, numbness, and tingling sensations (Lewis et al. 2013). Neuroimaging evidence demonstrated that non-painful PLP sensations have been associated with the activity of the posterior parietal cortex (Andoh et al. 2017; Bekrater-Bodmann 2022), thus linking PLP with abnormal multimodal integration processes following an amputation. Indeed, while a seamless cross-modal visual and tactile interaction supports a healthy embodiment, residual sensory information in the absence of visual cues may contribute to the development of PLP. However, the maintenance of the pre-existing body schema and PPS borders, which likely sustain PLP, can be used to boost the embodiment of a prosthesis (Anderson 2018; Bekrater-Bodmann 2022). As displayed in a recent study (Bekrater-Bodmann 2022), phantom limb sensations can support prosthesis embodiment in lower limb amputees, specifically when the phantom and prosthesis coexist within perceived space. As PPS reflects the spatial framework where this co-location might take place, a deeper understanding of the neural mechanisms behind PPS and its alterations after amputation could be crucial to address PLP and support prosthesis embodiment. Specifically, the development of biomimetic prostheses (Jarrassé et al. 2018; Touillet et al. 2018) based on the residual electromyographic activity associated with phantom limb movement could be fostered by additional knowledge on how phantom-mobility-based prostheses affect PPS.

### Stroke and hemispatial neglect

Stroke, a leading cause of long-term disability worldwide, displays several alterations in the experience of the body and in the representation of the space around the body. Indeed, brain damage derived from a stroke often results in a complex interplay between sensory, motor, and cognitive deficits that can alter the PPS representation.

Post-stroke lesions in the parietal cortex, particularly the posterior parietal cortex, affect the integration of sensory inputs from multiple modalities. Since the accurate processing of multisensory information is crucial to maintaining an updated representation of PPS, lesions in the parietal cortex can have a deep effect on PPS. These changes can manifest as challenges in perceiving the PPS boundaries, reduced awareness of objects within the PPS, or the capacity to effectively interact with the surrounding environment. In this regard, a recent study (Bassolino et al. 2022) examined the impact of upper limb sensorimotor deficit on PPS in 60 chronic stroke patients, highlighting that patients with lesions in the parietal operculum and frontoparietal white matter connections displayed reduced multisensory facilitation for stimuli presented around the affected limb (i.e., tactile and auditory stimuli). According to the authors, evidence of impaired PPS in individuals with sensorimotor impairment indicates a tight link between the integrity of the sensorimotor regions and PPS perception around the affected limb. In addition to the effects on sensorimotor functioning, a stroke is often associated with the development of neglect syndrome (Esposito et al. 2021). Often referred to as hemispatial neglect, this neuropsychological condition is often seen after right hemisphere lesions over several different cortical and subcortical areas and is characterized by an abnormal bias toward the space contralateral to the brain lesion. As a result, despite intact general motor and sensory functioning, individuals with neglect are unable to orient attention toward and attend to stimuli located in the contralesional space (Corbetta 2014). Taken together, these results strengthen the notion of the centrality of posterior parietal areas in mediating the perceptual and attentional aspects of PPS representation.

The neuropsychological tests used to evaluate the presence and severity of neglect, including drawing/coping tasks, line bisection, and target cancellation (Terruzzi et al. 2023), highlighted how neglect can variably affect the personal, peripersonal, and extrapersonal space perception. Although neglect following right hemisphere stroke is characterized by a generalized rightward bias, some patients display a dissociation between the perception of the peripersonal and extrapersonal contralesional space, thus supporting a distinct functional and neural separation in the processing of stimuli located in the peripersonal and external space (Buxbaum et al. 2004). A first account of the existence of different neural mechanisms behind the processing of peripersonal and external space information was provided by Previc (1998). Accordingly, the dorsal visual stream, which includes the inferior parietal cortex, is biased toward the processing of visuospatial information in the PPS, whereas the ventral pathway is mostly engaged in the processing of stimuli located in the external space (Previc 1998; Gilaie-Dotan 2016). The inferior parietal cortex plays a central role in this context, particularly in the integration of sensory and spatial information within PPS, as well as in the allocation of attention to objects near the body.

An investigation on postural imbalance highlighted that, differently from patients with extrapersonal neglect, patients with PPS neglect exhibited significant mediolateral center of pressure displacement (Nijboer et al. 2014). These results suggest that postural imbalance is not solely linked to visual neglect but also to the representation of the body and PPS. According to this study and the observed dissociation between PPS and extrapersonal neglect, it would be advisable to incorporate PPS representation assessment into the evaluation and treatment of neglect within stroke rehabilitation programs. This additional dimension may offer valuable insights for effectively managing this syndrome.

### Apraxia

Apraxia, a neurological disorder characterized by impaired motor planning and execution of learned skilled movements, presents a unique window into the relationship between higher-order cognitive processes and sensorimotor functions. Indeed, although patients with apraxia display intact motor and sensory functions, the difficulty in coordinating these primary functions to produce appropriate movements highlights the “higher-order cortical” nature of this disorder. As such, besides its hallmark motor deficits, apraxia also affects functions related to the perception and interaction with the immediate environment, including the PPS.

The few available studies on the representation of PPS in apraxia have largely focused on mirror apraxia, a condition resulting from damage to the posterior parietal cortex, in which patients display deficits in reaching objects presented through a mirror. Binkofski and colleagues (Binkofski et al. 2003) investigated whether mirror apraxia is similarly exhibited in the PPS and body space. Patients with lesions located around the inferior parietal lobule and mirror apraxia were asked to reach for objects presented through a mirror, either in PPS or body space. Results showed that, while reaching movements toward objects located in the body space was unaltered, performance declined when objects were positioned in the PPS, with patients reaching towards the mirror as if the objects were located on their body surface.

This intriguing observation led the authors to propose a potential dissociation between the representations of body schema and PPS in individuals with apraxia, with objects located on the body’s surface being more readily integrated into the body schema. Additional insights from positron emission tomography (PET) scans suggested that this deficit in reprogramming movements from the mirror to the real space may arise from lesions within the ventro-dorsal pathway, offering valuable insights into the neural basis of mirror apraxia in relation to PPS and body space (Binkofski et al. 2003). From a translational point of view, the dissociation between body schema and PPS may be leveraged to boost the recovery of patients suffering from apraxic syndromes. To date, most of the rehabilitation protocols proposed for apraxia lack a strong theoretical rationale (Worthington 2016). Aside with providing a theoretical framework to describe symptoms observed in apraxic patients, knowledge about the PPS and its relation to motor functions could help inform rehabilitation protocols.

### The emotional and social PPS: pain, trait anxiety, phobic and defensive behavior

Based on the evidence gathered on patients with sensorimotor impairment and physical disabilities, it has become apparent that there is an intricate relationship between embodiment, the perception of the space surrounding own’s body, and the ability to interact with the objects and living beings around us. Indeed, some emerging accounts suggest that the integration of exteroceptive and interoceptive information supports an integrated representation of the self as a separate entity from the environment and the others (Bogdanova et al. 2021). While the implications for one’s sense of embodiment, body schema, and perception of the self are apparent, PPS processing also supports functional interactions with the environment. First, despite being two different domains, the perception of the PPS and the psychological concept of “personal space” or “flight zone” are connected (Graziano and Cooke 2006). As previously highlighted, PPS representation also features nociceptive stimuli (De Paepe et al. 2017). The tight interaction between PPS and the nociceptive system has probably evolved to prompt immediate withdrawal from noxious stimuli directed to the face and body (Sambo et al. 2012; Cléry et al. 2015; Vieira et al. 2020). In particular, while the impact of acute pain in directly modulating the boundaries of PPS has been debated (Vittersø et al. 2019), alterations in PPS representation have been consistently observed in different chronic painful syndromes, including complex regional pain syndrome (CRPS) (Reid et al. 2016; Filbrich et al. 2017b; Bultitude et al. 2017), trigeminal neuralgia(Bufacchi et al. 2017) or episodic migraine (Ayas et al. 2020). The adaptations in the representation of PPS probably serve as defensive mechanisms, aimed at preventing contact or additional harm to hyperalgesic body parts. Considering the significant similarity between the brain’s representations of physical and emotional pain (Xiao and Zhang 2018), these mechanisms may have evolved from initially facilitating the avoidance of physical pain to eventually facilitating avoidance of perceived threats and, more broadly, avoidance mechanisms in general. Being the PPS the primary zone where immediate interactions with animate and inanimate entities occur, it certainly influences and is influenced by the subjective levels of comfort in different social and environmental contexts. While research has firmly established the influence of psychological and personality factors on the perception of interpersonal space, their impact on the perception of PPS has only recently been explored. As an example, a recent study highlighted how PPS boundaries may be affected by the social context and attachment type (von Mohr et al. 2023). According to their findings, securely attached individuals display a flexible separation between PPS and extrapersonal space based on the social context, with the PPS boundaries becoming sharper when strangers approach them. Conversely, individuals with high levels of anxious attachment displayed a less defined differentiation between PPS and extrapersonal space. This result is in line with neuroimaging evidence showing that the hyperactivation of the parieto-frontal network that monitors PPS is linked to anxious attachment (Nasiriavanaki et al. 2021). Further supported by evidence of expanded PPS in individuals with trait anxiety (Sambo and Iannetti 2013b), these results help to conceptualize the PPS not only as a space of action and reach but as a safety margin where individuals can be safely reached by external entities. This double function of the PPS, one action-oriented and one defensive-oriented, has been thoroughly explored by (de Vignemont and Iannetti 2015), highlighting the differences between these two functions in terms of their sensorimotor features. As Rabellino and colleagues recently addressed in a comprehensive review (Rabellino et al. 2020), the study of PPS representation has important implications for psychopathology. Along with trait anxiety being linked to larger PPS (Sambo and Iannetti 2013b; Iachini et al. 2015), a study recently found that individuals with high levels of claustrophobia and dog phobia showed larger PPS (Lourenco et al. 2011; Taffou and Viaud-Delmon 2014).

Overall, these studies suggest a link between phobic behavior and expanded PPS, possibly indicating that the security boundaries enclosed in the PPS may have a key role in the etiology of phobias. The relevance of PPS perception in the determinism of anxious symptomology may explain the increasing popularity and effectiveness of virtual-reality based treatments, which act by manipulating an artificial perception of objects within PPS boundaries (Boeldt et al. 2019).

In addition to the defensive function of PPS, in recent years there has been a substantial surge in research exploring the influence of PPS on social abilities, with a particular emphasis on conditions such as autism and schizophrenia.

### Schizophrenia

Schizophrenia is a complex and chronic mental health disorder that, among the numerous behavioral, cognitive, and emotional dysfunctions, is also characterized by body image alterations, a weakened sense of self, and blurred self-other differentiation.

Individuals with schizophrenia tend to be more susceptible to experiencing distortions in bodily self-perception, as demonstrated in phenomena like the Rubber Hand Illusion(Thakkar et al. 2011) and the Pinocchio Illusion (i.e., perceiving the nose as elongated after specific sensory stimulation) (Michael and Park 2016). These observations prompted researchers to hypothesize that the disrupted self-others distinction may be related to difficulties in representing the PPS, which serves as the boundary zone between the self and the external world. The experimental evidence directly assessing PPS representation in the schizophrenic spectrum has only started to accumulate, yielding diverse and sometimes contradictory findings (Noel et al. 2017; Di Cosmo et al. 2018; Paredes et al. 2020; Lee et al. 2021b; Ferroni et al. 2022).

A first working model of PPS alterations in schizophrenia (Noel et al. 2017) proposed that schizophrenic patients display blurred boundaries between the PPS and extrapersonal space and that multisensory stimulation could help reestablish normal PPS boundaries and improve social interactions. This initial hypothesis was challenged by Di Cosmo and colleagues with evidence of a sharper separation between PPS and extrapersonal space in individuals with schizophrenia and high schizotypy features, as indicated by the faster transition between the PPS and external space (Di Cosmo et al. 2018). Furthermore, it was observed that the reduced PPS was inversely linked to the intensity of negative symptoms, indicating a potential association between PPS representation and “withdrawal” symptoms, such as social isolation and avolition. A recent investigation confirmed that the size and boundary demarcation of PPS were negatively associated with negative symptoms, experiences of existential reorientation (self-disorder), and traits of schizophrenic autism (Lucarini et al. 2023). Remarkably, the collected PPS parameters were able to predict early and adult-onset schizophrenia subgroups, thus suggesting the relevance of PPS for the diagnosis of schizophrenia and for addressing its symptoms. Since the PPS acts as a crucial “buffer zone” between our intrapersonal and extrapersonal realms, the diminishment of PPS boundaries, as seen in individuals with schizophrenia, may limit their ability to perform voluntary actions and interact effectively with the world, but may also blur the distinction between self and others. Interestingly, another investigation found that PPS is modulated by the context in schizophrenic patients (Lee et al. 2021b). By employing a virtual reality-based visuotactile paradigm with social and nonsocial environments, the study confirmed previous accounts of overall reduced PPS in schizophrenic patients. However, the boundary between the self and the others was weaker during social but not in nonsocial contexts, thus highlighting the context-dependent and malleable nature of PPS representations. A narrower PPS extent and weaker self-other distinctions were also confirmed by Ferroni and colleagues (Ferroni et al. 2022). Importantly, based on the evidence accumulated on the extension of PPS with tool and prosthetic use, as well as the importance of multisensory integration for the plasticity of the PPS, the study also demonstrated that is possible to address PPS abnormalities in schizophrenic patients. Indeed, after completing a sensorimotor training in which participants manipulated small objects positioned in the extrapersonal space with a tool, the study observed an expansion and improved demarcation of PPS boundaries.

Altogether, current research provides additional support for the aberrations in spatial self-perception in schizophrenia patients. This helps shed light on the origins of its subjective aspects and enables a better understanding of the fundamental abnormalities that may give rise to more intricate symptoms.

Furthermore, the involvement and manipulation of PPS perception may also provide an operational framework for virtual-reality-based intervention, as a strategy to improve social avoidance and momentary paranoid ideation in social scenarios in schizophrenic patients (Pot-Kolder et al. 2018).

Indeed, virtual reality environments can be designed to simulate real-life scenarios, allowing individuals to practice and improve their PPS perception in a controlled setting. Specifically, in addition to task-specific training involving spatial awareness, such as reaching for objects, and navigating through spaces, virtual reality therapy can provide a platform for practicing social interactions, allowing individuals to navigate PPS norms and improve their social skills in an environment tailored to the individual’s needs.

In conclusion, the existing literature underscores the potential significance of PPS in the etiology of some distinct features of schizophrenia and how addressing such PPS abnormalities with targeted interventions (e.g., multisensory integration training or virtual reality) holds promise as a viable treatment option for this chronic and debilitating disorder.

### Autism spectrum disorder

Autism Spectrum Disorder (ASD) is a heterogeneous neurodevelopmental disorder characterized by a wide range of symptoms including behavioral, sensorial, and social impairment. Although the hallmark of ASD is a persistent deficit in engaging in social interactions, it is worth noting that a formal diagnosis of ASD also requires the presence of restricted or repetitive behaviors or activities (American Psychiatric Association 2013). Atypical sensory processing in ASD individuals has been extensively documented for a wide range of domains, including auditory, touch, vision, and social stimuli (Marco et al. 2011; O’Connor 2012; Nomi and Uddin 2015; DeBoth and Reynolds 2017; Balasco et al. 2020; Chung and Son 2020; Iannuzzo et al. 2022). Such sensory processing differences can pose challenges for individuals with ASD in effectively processing and integrating sensory information, thereby likely impacting their ability to comprehend and respond appropriately to stimuli within their PPS. Relevant work on the impact of sensory integration deficits on PPS found reduced body illusions in ASD individuals after virtual reality-based (Mul et al. 2019) and rubber hand illusion protocols (Cascio et al. 2012). Further, PPS was markedly smaller and characterized by sharper boundaries (Noel et al. 2017, 2020; Mul et al. 2019). Mul and colleagues experimentally investigated the relationship between PPS perception and bodily self-consciousness based on differences in multisensory processing (Mul et al. 2019). Their findings revealed that, in contrast to neurotypical individuals, participants with ASD demonstrated diminished full-body illusion, characterized by a reduced sense of self-identification and a limited shift in self-location. Interestingly, the strength of self-identification was found to have a negative correlation with the severity of autistic traits while positively contributing to empathy scores. Moreover, alike to schizophrenic individuals, ASD was associated with a notably smaller PPS representation with sharper boundaries. A similar effect was also observed in ASD children subjected to the rubber hand illusion (Cascio et al. 2012). Interestingly, despite an initial reduced effect of the illusion, after six minutes, ASD children displayed the same levels of illusion experience as healthy controls, and the degree of identification with the artificial body part was positively associated with empathy.

These important findings highlight that (1) ASD individuals’ difficulty in integrating the external body may be linked to delayed multisensory integration and (2) the malleability of the personal and peripersonal space has a role in shaping the interactions of the individual with the surrounding environment. In a series of studies (Candini et al., 2020, 2019), Candini and colleagues claimed that space regulation deficits in ASD only affect interpersonal interactions and not peripersonal, action-oriented, space. However, given the strong functional relationship between PPS representation, body schema, and interpersonal interactions, such dissociation may reflect the existence of different crossmodal integration mechanisms enabling the interactions with tools and other individuals. Although additional evidence is needed to explore how PPS affects the individual’s social and nonsocial interactions with the environment, a recent theoretical framework proposed that plastic shaping of bodily and PPS representations supports interpersonal motor interactions (Fanghella et al. 2021). Evidence of reduced empathy levels when barriers were placed in the PPS and a positive association between the activity of the motor, premotor, and sensorimotor areas, and empathy (Schiano Lomoriello et al. 2023) support the concepts of embodied social cognition and mirror neuron system. In this context, the PPS, our space for action, may malleably define the capacity to be permeated by the external world and our ability to interact with it.

Further research is essential to deepen our understanding of how intra-personal, peripersonal, and interpersonal spaces, and their flexible interactions may underlie the diverse sensory experiences and social interaction in typical and atypical neural development. By understanding the underlying mechanisms of PPS perception and social interaction difficulties in autism, clinicians can develop more effective treatments. This could include therapies that focus on improving PPS representation and social interaction skills, as well as therapies that target the underlying neural systems.

## Conclusions

The present work aimed at providing a comprehensive overview of the neuroanatomical correlates of the PPS processing, connecting the dots from both animal and human studies. Although many inconsistencies and controversies still divide us from a complete characterization of PPS encoding in the human brain, a few final considerations can be drawn:

- Early investigations on PPS encoding in the primate brain have led to the identification of a series of parallel fronto-parietal circuits underlying specific aspects of PPS encoding and representation.
- The main features of such circuitry, which here we define as the “core PPS network”, include the presence of multi-sensory input channels, the integration of sensory information into a space definition centered on specific body parts, and the direct, preferential link between elements included in this spatial representation and their behavioral correlates, such as grasping, defence mechanisms or eye movements; through this circuitry, a direct correspondence between spatial representation and complex behavior is established.
- Neuroimaging evidence in the human brain has mostly confirmed these results, highlighting the relevance of premotor and parietal areas in mediating different aspects of PPS processing. Data-driven fMRI investigations of brain circuitry at rest have underlined how these structures all belong to a common network, the so-called “dorsal attention network”. This network, which also involves other brain regions, allows the representation of the environment in terms of priority and relevance for action.
- Human fMRI studies have also clarified the role of other brain structures in mediating specific aspects of PPS representation. Such regions include the supramarginal gyrus, anterior insula, anterior cingulate cortex, and subcortical areas such as the cerebellum, amygdala, pulvinar, or superior colliculi. Some of these regions were shown to be part of a so-called “salience” or “ventral attention network” which mediates attentional response to emotionally or behaviorally relevant stimuli.
- Disturbances in PPS perception have been associated with deficits in movement planning, body ownership, spatial awareness, and social interactions, thus underlying the paramount importance of PPS in maintaining motor control, spatial awareness, and adaptive social behaviors. Clinicians may take advantage of translational neuroimaging research to understand how PPS dysfunction can lead to various symptoms paving the way for more detailed insights into the role played by the PPS network in the pathophysiology of different neuropsychiatric disorders.
- Finally, from a clinical and translational viewpoint, the PPS network can represent a possible target for management and therapeutic intervention, as multiple rehabilitation strategies, ranging from motor imagery to virtual reality, can manipulate and alter the perception of PPS and the representation of its boundaries.

Taken together, these findings further confirm the relevance of PPS representation for cognitive, emotional, and social functions and further delineate an “extended PPS network”, which ultimately relies on the interactions between distinct dorsal and ventral networks for spatial attention, emotion, and cognition.

While the rise of data-driven, large-scale neuroimaging has provided further advances in our knowledge of the circuitry underlying PPS processing in the brain, on the other hand, these findings leave room for further investigations. Resting-state fMRI investigations have clarified the network structure of cortical and subcortical nodes involved in PPS processing: in this perspective, a possible step forward may involve investigating the relationship between architectural features of these networks (e.g., as derived from graph analysis or dynamic causal modeling) and behavioral measures of PPS processing. On the clinical side, the development of standardized, simple, and reliable tools for PPS assessment could further ease the investigation of relationships between PPS and anatomical and functional measures. Finally, improved knowledge of the network anatomy of PPS processing in the brain could inform rehabilitation protocols for neuropsychiatric conditions that involve PPS distortions, opening the field to the perspective of designing specific targets for non-invasive brain stimulation methods, such as transcranial magnetic stimulation or transcranial direct current stimulation (Bassolino et al. 2018; Vergallito et al. 2019).

Finally, some limitations of the present work must be acknowledged. Given the broad topic covered by the present review, which ranges from anatomical and physiological investigation in primates to retrospective analysis in clinical populations, a narrative approach was preferred. This choice implies that the analysis of the existing literature was not performed with a systematic approach, opening the way to possible bias in selecting and reporting the results. This is particularly relevant for the section discussing PPS alteration in different clinical populations, that may have benefited from a systematic approach to the existing literature. While a systematic review or meta-analysis of PPS alterations in a specific clinical population goes beyond the aims of the current work, the Authors warrant further investigations to clarify and characterize PPS alterations observed in clinical conditions, as well as their role in rehabilitation protocols.

In conclusion, a better understanding of the anatomical underpinnings of PPS representation may represent a relevant step towards a better understanding of multiple higher-order cognitive functions and their derangements.

## Data Availability

Online sources including PubMed/MEDLINE, Google Scholar, EMBASE have been used for the purpose of the review.
